# Analysis of risk factors for complications after laparoscopic cholecystectomy

**DOI:** 10.1016/j.heliyon.2023.e18883

**Published:** 2023-08-03

**Authors:** Jing-nan Fu, Shu-chang Liu, Yi Chen, Jie Zhao, Tao Ma

**Affiliations:** a Department of Minimally Invasive Surgery, Characteristics Medical Center of Chinese People Armed Police Force, Tianjin, China; b Department of General Surgery, Tianjin Medical University General Hospital, Tianjin, China; c Department of Intensive Care Unit, Tianjin Medical University General Hospital, Tianjin, China

**Keywords:** Laparoscopic cholecystectomy, Complication, Risk factors

## Abstract

To analyze the risk factors of complications after laparoscopic cholecystectomy in 478 patients in our hospital. Methods: The clinical data of 478 patients who underwent laparoscopic cholecystectomy in our hospital from March 2018 to September 2022 were collected, and the occurrence of postoperative complications and related risk factors were analyzed. Results: A total of 36 patients (7.53%) had complications, including 9 cases (1.88%) of abdominal hemorrhage, 8 cases (1.67%) of bile duct injury, and 19 cases (3.97%) of biliary fistula. Univariate analysis showed that adhesions of Calot triangle, anatomical variation and gallbladder wall thickness greater than 5 mm were associated with postoperative complications (all P < 0.05). Multivariate analysis showed that: Calot triangle adhesion (OR = 3.041, 95%CI = 1.422–6.507), anatomical variation (OR = 4.368, 95%CI = 1.764–10.813) and gallbladder wall thickening (OR = 2.827, 95%CI = 1.422–6.507). 95%CI = 1.274–6.275) were independent risk factors for complications after laparoscopic cholecystectomy (all P < 0.05). Conclusion: In order to reduce the occurrence of postoperative complications, the risk factors of LC should be well understood and the preoperative preparation should be made.

## 1 Introduction

Laparoscopic cholecystectomy (Laparoscopiccholecystectomy, LC) is a common form of minimally invasive surgery, is the first selection of gall bladder calculi surgery, than with laparotomy with little trauma and quick recovery, small painful, eat early, etc [1]. In recent years, with the gradual relaxation of the surgical indications of laparoscopic cholecystectomy, the number of surgical cases is also increasing, and the corresponding surgical complications are also increasing, mainly including: abdominal bleeding, biliary fistula, abdominal infection, bile duct injury and organ injury [2]. Previous studies have found that postoperative complications of LC may be related to Calot triangle adhesion, anatomical variation of biliary tract structure, thickening of gallbladder wall, history of abdominal surgery, number of gallstones and gallbladder atrophy. If preoperative preparation is not made in time, the above complications are likely to occur after surgery, and severe cases can lead to death [3]. Therefore, it is of great clinical significance to explore the risk factors of postoperative complications of LC.

Based on the study of postoperative complications of LC in our hospital, the connection between postoperative complications and risk factors such as adhesion of Calot triangle, variation of biliary anatomy, thickness of gallbladder wall, history of abdominal surgery, multiple stones of gallbladder and atrophy of gallbladder were analyzed, so as to formulate more complete perioperative management measures.

## 2 Materials and methods

### 2.1 General information

We report that surgical studies follow the STROCSS(Enhanced Surgical Cohort Study Reports) guidelines. The clinical data of 478 patients who underwent LC in our hospital from March 2018 to September 2022 were collected. All patients had acute cholecystitis. The diagnostic criteria of cholecystitis mainly include symptoms, signs, laboratory examination and imaging examination. Cholecystitis may be indicated if the patient has right upper abdominal pain, nausea, vomiting, fever and other symptoms accompanied by right upper abdominal tenderness, muscle tension, positive Murphy sign and other physical signs, as well as abnormal blood routine, liver function, gallbladder ultrasound and other tests.

The inclusion criteria were as follows: all patients aged ≧18 years old, diagnosed with gallbladder stones by preoperative CT and color Doppler ultrasound, all patients were admitted in emergency, preoperative examination was completed, and LC surgery was performed in emergency. Exclusion criteria: age <18 years old; Patients with hematological diseases, malignant tumors, hepatic and renal insufficiency, severe infectious diseases, septic shock, pregnancy and lactation.

### 2.2 Surgical methods

All operations were performed under general anesthesia with oral intubation, routine disinfection, drape, pneumoperitoneum (CO_2_ pressure was adjusted at 12–14 mmHg), and the operation position was set at 30° on the left side. A 1 cm Trocar was drilled above the umbilicus and under the xiphoid process, and a 0.5 cm Trocar was drilled under the right costal margin and at the mid-clavicular line. Laparoscopy and corresponding surgical instruments were placed in each Trocar hole. The internal organs and infection in the abdominal cavity, adhesion of the gallbladder triangle, thickening of the gallbladder wall, and variation of the gallbladder and cystic duct were explored. The cystic duct and the cystic artery were dissected out and clamped with Hemorock respectively. The gallbladder bed was separated, and the gallbladder was completely removed by antegrade, retrograde or combined antegrade and retrograde methods. Then the gallbladder was removed from the puncture hole under the xiphoid process.

### 2.3 Observation indicators

The postoperative general conditions of the patients were recorded, and the occurrence of postoperative complications was observed and recorded. At the same time, the operation videos and photos were collected and the operation records were written. The thickness of the gallbladder wall (the patient's gallbladder thickness was determined by ultrasound examination), the number of gallstones, whether the gallbladder was atrophic, the adhesion of the Calot triangle and the anatomical variation of the Calot triangle were objectively recorded, and the clinical data of the patients were kept. To analyze the risk factors of postoperative complications after LC and determine their connection with complications.

The degree of adhesion of the gallbladder triangle: the patient has repeated inflammation of the gallbladder, especially the incarceration of gallbladder duct and gallbladder neck stones. Due to long-term stimulation of inflammation, the surrounding tissues are also affected by the inflammation of the gallbladder wall, resulting in the dense adhesion of the gallbladder triangle which is hard and difficult for intraoperative separation.

### 2.4 Statistical methods

SPSS 20.0 statistical software was used for data analysis, and the count data were expressed as percentage. The type of logistic regression used is backward Wald. Chi-square test and multivariate Logistic regression analysis were used for data analysis, and P < 0.05 was considered statistically significant.

## 3 Results

### 3.1 General information of the patients

Among the 478 patients undergoing LC, 36 patients had complications (7.53%), including 9 patients with abdominal hemorrhage (1.88%) and 2 patients treated by surgery. Bile duct injury occurred in 8 cases (1.67%), and 2 cases were treated by surgery. Biliary fistula occurred in 19 cases (3.97%), and 4 cases were treated by surgery. (See Table 1 for details).

**Table 1:** Basic data of patients with complications（n）.

Complications	n	Male	female	age	Conservative treatment	Surgical treatment
Intra-abdominal hemorrhage	9	5	4	36.900 ± 12.60（21–57）	7	2
Bile duct injury	8	4	4	38.904 ± 18.10（19–67）	6	2
Biliary fistula	19	11	8	47.381 ± 15.07（19–73）	15	4

### 3.2 Univariate analysis of risk factors related to complications after laparoscopic cholecystectomy

**Fig. 1:**
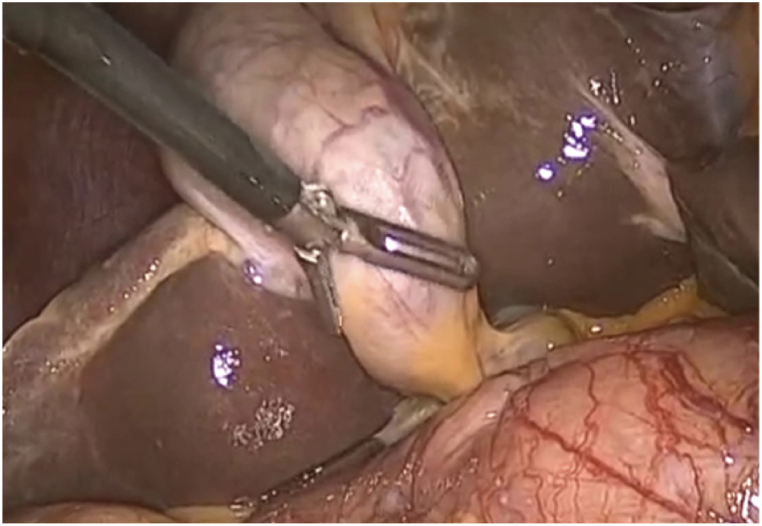
In suppurative cholecystitis, the wall of the gallbladder was thickened, the tissue was hyperemic and edematous, and the anatomy of the gallbladder triangle was unclear.

Univariate analysis showed that: Among the above risk factors, Calot triangle adhesion, anatomical variation and gallbladder wall thickening more than 5 mm were all related to the occurrence of postoperative complications after LC, and the differences were statistically significant (P < 0.05) (Fig. 1). However, there was no significant difference in the connection between postoperative complications of LC and history of abdominal surgery, whether the gallbladder was multiple stones or not, and whether the gallbladder was atrophic or not (P > 0.05) (Table 2).

**Table 2:** Univariate analysis of risk factors for postoperative complications after LC.

Factors	n	Complications	χ2	P
Variation of anatomy				
Yes	36	7（19.44%）	6.192	0.013
No	442	29（6.56%）		
Atrophy of the gallbladder				
Yes	43	4（9.30%）	0.025	0.874
No	435	32（7.36%）		
History of abdominal surgery				
Yes	48	6（12.50%）	1.182	0.277
No	430	30（6.98%）		
Gallbladder wall thickness				
>5 mm	78	11（14.10%）	5.779	0.016
≦5 mm	400	25（6.25%）		
Number of stones				
Multiple cases	198	16（8.08%）	0.147	0.702
single lesion	280	20（7.14%）		
Calot's triangle adhesions				
Yes	96	13（13.54%）	6.231	0.013
No	382	23（6.02%）		

### 3.3 Multiple logistic regression analysis of risk factors related to complications after laparoscopic cholecystectomy

According to the results of the above research, the complications of patients undergoing laparoscopic cholecystectomy were taken as factor variables, and the three statistically significant indicators in the results of univariate analysis: anatomical variation, gallbladder wall thickening and Calot's triangle adhesion were taken as independent variables. They were included in the Logistic regression model for multivariate analysis. Anatomical variation, gallbladder wall thickening, and Calot triangle adhesion were still risk factors for surgical complications of LC, and the differences were statistically significant (P < 0.05), as shown in Table 3.

**Table 3:** Multivariate Logistic regression analysis of risk factors for complications after laparoscopic cholecystectomy.

Risk factors	B	SE	Wald	P	OR	95%CI
Calot triangle adhesion	1.112	0.388	8.216	0.004	3.041	1.422–6.507
Anatomical variation	1.474	0.463	10.159	0.001	4.368	1.764–10.813
Gallbladder wall thickness	1.039	0.407	6.525	0.011	2.827	1.274–6.275

## 4 Discussion

Cholecystolithiasis is the most common biliary tract disease. Clinically, it is mainly divided into asymptomatic cholecystolithiasis and symptomatic cholecystolithiasis. Symptomatic gallstones should be treated in time, and the main clinical manifestations are abdominal pain, abdominal distension, nausea, vomiting, jaundice, fever and so on. In severe cases, gallbladder perforation, intestinal obstruction, sepsis and other serious complications can be life-threatening, so it is necessary to perform surgical treatment in time [4]. In the past, the main method was mainly open surgery. At present, with the development of minimally invasive technology, abdominal cholecystectomy has been carried out more and more, and its surgical indications are wider and wider. The length of hospital stay is short and easy to accept. It has become the preferred surgical method for the treatment of gallstones [5]. In addition, with the use of advanced hemostatic surgical instruments such as ultrasonic scalps and hemostatic clips, the bile ducts and blood vessels can be carefully operated, and the amount of bleeding during surgery can be further reduced [[6], [7], [8], [9]]. Although laparoscopic cholecystectomy has many advantages, there are still many postoperative complications, mainly including: abdominal bleeding, biliary fistula, abdominal infection, bile duct injury and organ injury [10]. A total of 478 patients with gallstones in our hospital were collected in this study. All patients underwent LC, and 36 of them had postoperative complications, with an incidence of 7.53%. There were 9 cases of abdominal hemorrhage (1.88%), 8 cases of bile duct injury (1.67%), and 19 cases of biliary fistula (3.97%). It is suggested that we should carefully examine patients during the perioperative period before operation, understand the risk factors of patients during the perioperative period, and carry out early intervention and prevention, so as to reduce the occurrence of postoperative complications and improve the prognosis of patients.

The results of univariate analysis of this study showed that anatomical variation, gallbladder wall thickening and Calot triangle adhesion were related to the occurrence of postoperative complications after LC, and the difference was statistically significant (P < 0.05). The above three risk factors were further analyzed by Logistic regression, and the results showed that the above three risk factors were still the related risk factors affecting the postoperative complications of LC, and the difference was still statistically significant (P < 0.05). The anatomical variation of the biliary tract makes it difficult for the surgeon to determine the correct anatomical position of the cystic duct, common bile duct and cystic artery. In addition, the local inflammation of gallstones causes unclear anatomical relationship, so postoperative complications such as bleeding and biliary fistula are easy to occur. Calot's triangle is a triangular region formed by the common hepatic duct, cystic duct and the lower edge of the liver, which includes part of the right hepatic artery and cystic artery. Biliary tract infection and inflammatory exudation can cause adhesion in this region, resulting in unclear anatomy. It is difficult to accurately find the cystic duct and cystic artery during operation, which is easy to damage the right hepatic artery and cause abdominal hemorrhage. In severe cases, right hepatic ischemia can occur [[11], [12], [13]]. The thickening of the gallbladder wall indicates the presence of inflammation in the gallbladder, and the more obvious the thickening of the gallbladder wall indicates the more serious the congestion and edema of the gallbladder wall. The thickened gallbladder wall can cause adhesion of the gallbladder to the surrounding tissues, resulting in unclear anatomical layers, and easy to cause complications such as gallbladder rupture, bile duct injury, and surrounding organ injury.

It should be noted that there are still limitations in the present study. Complications after cholecystectomy include bile duct injury, bile leakage, bile duct stricture, complicated cholangitis, intraperitoneal bleeding, abdominal effusion, abdominal infection, liver injury, gastric antrum and duodenum injury, colon injury, laparoscopic pneumoperitoneum puncture, gas embolism, lower limb vein thrombosis, incision infection, incision dehiscence, etc. The number of complications studied in this study is limited, and risk factors for other complications will be further explored in the later study.

In conclusion, anatomical variation, thickness of gallbladder wall, and adhesion of Calot triangle are risk factors for postoperative complications of LC. Therefore, we should be highly alert to the occurrence of complications in patients undergoing LC, and the patient's condition should be mastered in detail before operation. The vital signs of patients should be closely observed after operation, and the abdominal color Doppler ultrasound, liver and kidney function, electrolytes and blood routine should be reviewed in time to find abnormal results and treat them as soon as possible.

## Data availability statement

All data generated or analyzed during this study are included in this paper.

## Author contribution statement

Jing-nan Fu: Analyzed and interpreted the data; Wrote the paper.

Tao Ma: Contributed reagents, materials, analysis tools or data.

Shu-chang Liu: Conceived and designed the experiments.

Yi Chen: Performed the experiments.

Jie Zhao: Analyzed and interpreted the data.

## Additional information

No additional information is available for this paper.

## Declaration of competing interest

We declare that we have no financial and personal relationships with other people or organizations that can inappropriately influence our work, there is no professional or other personal interest of any nature or kind in any product, service and/or company that could be construed as influencing the position presented in, or the review of, the manuscript entitled.

Our research includes experimentation on human subjects, which have approvaled by our institute's ethics committee prior to conducting the research, we have stated confirmation that these experiments were conducted according to established ethical guidelines, and informed consent obtained from the participants. The name of the Ethics Committee is ‘Characteristics Medical Center Ethics Committee of the Chinese People's Armed Police Force’. And the Ethics approval number is 2023-0020.

Any patient who appears in our publication has given consent for their images to be published.
